# Hydrogen-rich saline alleviates early brain injury via reducing oxidative stress and brain edema following experimental subarachnoid hemorrhage in rabbits

**DOI:** 10.1186/1471-2202-13-47

**Published:** 2012-05-15

**Authors:** Zong Zhuang, Meng-liang Zhou, Wan-chun You, Lin Zhu, Chi-yuan Ma, Xue-jun Sun, Ji-xin Shi

**Affiliations:** 1 Department of Neurosurgery, Jinling Hospital, School of Medicine, Nanjing University, 305 East Zhongshan Road, Nanjing, 210002, Jiangsu Province, China; 2 Department of Diving Medicine, Faculty of Naval Medicine, Second Military Medical University, Shanghai, 200433, China

**Keywords:** Subarachnoid hemorrhage, Hydrogen, Oxidative stress, Brain edema, Early brain injury

## Abstract

**Background:**

Increasing experimental and clinical data indicate that early brain injury (EBI) after subarachnoid hemorrhage (SAH) largely contributes to unfavorable outcomes, and it has been proved that EBI following SAH is closely associated with oxidative stress and brain edema. The present study aimed to examine the effect of hydrogen, a mild and selective cytotoxic oxygen radical scavenger, on oxidative stress injury, brain edema and neurology outcome following experimental SAH in rabbits.

**Results:**

The level of MDA, caspase-12/3 and brain water content increased significantly at 72 hours after experimental SAH. Correspondingly, obvious brain injury was found in the SAH group by terminal deoxynucleotidyl transferase-mediated uridine 5’-triphosphate-biotin nick end-labeling (TUNEL) and Nissl staining. Similar results were found in the SAH + saline group. In contrast, the upregulated level of MDA, caspase-12/3 and brain edema was attenuated and the brain injury was substantially alleviated in the hydrogen treated rabbits, but the improvement of neurology outcome was not obvious.

**Conclusion:**

The results suggest that treatment with hydrogen in experimental SAH rabbits could alleviate brain injury via decreasing the oxidative stress injury and brain edema. Hence, we conclude that hydrogen possesses the potential to be a novel therapeutic agent for EBI after SAH.

## Background

Subarachnoid hemorrhage (SAH), accounting for about 5-7% of all strokes, is a frequently seen devastating disease with high mortality and disability [1]. After decades of studies both in basic and in clinic, considerable advances have been made in diagnostic methods, endovascular techniques, and surgical and medical therapy. Nonetheless, the mortality and morbidity rates after spontaneous SAH have not been decreased [2], the outcome of the patients with SAH remains poor, with the mortality rate as high as 45% and high morbidity among survivors [3,4]. As a result, there has been a consistent interest in understanding the molecular mechanisms responsible for SAH.

Two main issues have been focused following SAH: vasospasm and early brain injury (EBI). It was thought that cerebral ischemia and other related complications, which result in poor outcome, were more or less exclusively caused by arterial narrowing after SAH. Hence, vasospasm has been the focus of the majority of research efforts during the past decades on SAH. Unfortunately, little success has been achieved in improving outcome following SAH [5,6]. Especially, this idea has recently been challenged by the failure of the drug clazosentan to improve patients’ outcome, despite reversing vasoconstriction [6,7]. Therefore, we propose to look beyond vasoconstriction, and consider more factors, such as ischemia, disruption of the blood–brain barrier, activation of apoptotic and inflammatory pathways, and cortical spreading depression in the early stage following SAH [8,9].

Increasing evidence has proved that the EBI period, encompassing 24–72 hours following SAH, is closely bound up with the poor prognosis [10,11]. Moreover, numerous studies have demonstrated that oxidative stress and brain edema are critical in the development of early brain injury after SAH [12-14]. Among the complex factors involved in the neuronal death after SAH, oxidative stress has been consistently highlighted. Reactive oxygen species (ROS) or reactive nitrogen species (RNS) (such as the hydroxyl radical (·OH), superoxide anion (·O^-^_2_·), hydrogen dioxide (H_2_O_2_), nitric oxide (NO) and peroxynitrite (ONOO^−^)) have been believed to play a critical role following SAH [15].

Brain edema has been proved to be a major component of EBI as a direct consequence of the disruption of the blood brain barrier (BBB) [16,17], rather than the result of vasospasm [13]. In addition, global edema has been considered as an independent risk factor for mortality and poor outcome after SAH [13]. It has been shown that approximately 8% of patients had global cerebral edema detected by CT scan on admission, and approximately additional 12% developed appreciable edema over the first 6 days [13]. Meanwhile, mounting evidence suggests that oxidative stress injury is intimately associated with brain edema [18].

Given the substantial evidence for significant levels of oxidative stress and brain edema following SAH, the use of free radical scavengers is a reasonable approach to improve the prognosis. Hence, hydrogen (H_2_), which has been gradually recognized as a potent anti-oxidative and anti-apoptotic agent [19-21], may act as an effective therapeutics after SAH. Many studies have evinced that H_2_ possesses cytoprotective effects in different cell types and disease models, including ischemia-reperfusion injury [22], drug toxicity [23] and trauma [24]. All these findings make H_2_ a potential perfect candidate to provide protective effect for the brain after SAH. Hence, in this study, we used an SAH model in rabbits to test whether H_2_ therapy could ameliorate EBI following SAH.

## Methods

### Animal preparation

The experimental protocol of using animals was approved by the Animal Care and Use Committee of Nanjing University and conformed to the Guide for the Care and Use of Laboratory Animals from the National Institutes of Health. All New Zealand white rabbits used in the present study were purchased from Experimental Animal Central of Jinling Hospital (Nanjing, China). They were acclimated in a humidified room and maintained on the standard pellet diet at the Animal Center of Jinling Hospital for 10 days before the experiment. The temperature in both the feeding room and the operation room was maintained at about 25°C.

### Hydrogen-rich saline preparation

The hydrogen-rich saline was prepared as described previously [25]. Briefly, H_2_ was dissolved in normal saline for 2 h under high pressure (0.4 MPa) to the supersaturated level(more than 0.6 mmol/L) by using a self-designed hydrogen-rich water-producing apparatus provided by the Department of Diving Medicine, the Second Military Medical University, China. The saturated hydrogen saline was stored under atmospheric pressure at 4°C in an aluminum bag with no dead volume and was sterilized by gamma radiation. Hydrogen-rich saline was freshly prepared every week to ensure a constant concentration. Gas chromatography was used to confirm the content of hydrogen in saline by the method described by Ohsawa et al. [20].

### Experimental design

Adult male New Zealand white rabbits weighing from 2.4 to 2.8 kg were assigned randomly to four groups: (1) control group (n = 18), (2) SAH group (n = 18), (3) SAH + saline group (n = 18), and (4) SAH + hydrogen-rich saline group (n =18). In the rabbits of SAH + hydrogen-rich saline group, hydrogen-rich saline (5 ml/kg) [25] was administered immediately after the first blood injection, which continued every 12 hours for 72 hours. Rabbits of SAH + saline group received equal volumes of saline at corresponding time points. Both hydrogen-rich saline and saline were injected intraperitoneally. Six rabbits in each group were killed with the fixation perfusion method. The brains were taken for Nissl staining, and TUNEL staining. The other six rabbits in each group were exsanguinated and decollated, with the brains removed and frozen in liquid nitrogen immediately for biochemical studies. The rest of the six were exsanguinated and decollated, and the brains were used for brain water content measurement.

### Two-hemorrhage rabbit model

Experimental SAH was produced according to the two-hemorrhage method [26]. The rabbits were anesthetized with an intramuscular injection of a mixture of ketamine (25 mg/kg) and droperidol (1.0 mg/kg) on day 0. Under spontaneous breathing, a 23-gauge butterfly needle was inserted percutaneously into the cistern magna. Then about 1.5 ml nonheparinized fresh autologous auricular arterial blood was slowly injected into the cisterna magna for 1 minute under aseptic technique. Then animals were kept in a 30° head-down position for 30 minutes. After recovery from anesthesia, they were returned to the feeding room. Forty-eight hours after the first SAH, the second injection was performed in the same manner as the first.

### Clinical evaluation

Clinical scores were recorded based on independent observations by a veterinarian who was blinded to the study. The scores of animals sacrificed on day 3 were recorded daily by using the modified scoring table (Table 1) as described previously [27].

**Table 1:** Behavior scores

**Category**	**Behavior**	**Score**
	Finished meal	**0**
**Appetite**	Left meal unfinished	**1**
	Scarcely ate	**2**
	Active, barking or standing	**0**
**Activity**	Lying down, will stand and walk with	**1**
	some stimulation	
	Almost always lying down	**2**
	No deficits	**0**
**Deficits**	Unable walk due to ataxia or paresis	**1**
	Impossible to walk and stand due to ataxia and paresis	**2**

### Perfusion-fixation

The rabbits scheduled for death were anesthetized with an intramuscular injection of a mixture of ketamine (40 mg/kg) and droperidol (2.5 mg/kg). The animals were then intubated endotracheally with a 3.5 mm diameter tracheal tube and mechanically ventilated with a rodent ventilator (SGC, China). Perfusion-fixation was then performed. The thorax was opened with a cannula placed in the left ventricle, the descending thoracic aorta clamped, and the right atrium open. Perfusion was begun with 500 mL of physiological phosphate buffer solution (PBS, pH 7.4) at 37°C, followed by 500 mL of 10% buffered formaldehyde under a perfusion pressure of 120 cm H_2_O. After perfusion fixation, the whole brain was removed and immersed in the same fixative solution.

### Nissl staining and cell counting

For Nissl staining, the 4 μm sections were hydrated in 1% toluidine blue at 50°C for 20 min. After rinsing with double distilled water, they were dehydrated and mounted with permount. Normal neurons have relatively big cell body, rich in cytoplasm, with one or two big round nuclei. In contrast, damaged cells show shrunken cell body, condensed nuclei, dark cytoplasm, and many empty vesicles. Cell counting was restricted to the external granular lamina layer of the occipital cortex. Six random high power fields (x400) in each coronary section were chosen bilaterally, and the mean number of surviving neurons in the six views was regarded as the data of each section. Every third coronary section starting from 3.0 mm posterior to the optic chiasma was collected and a total of four sections from each animal were used for quantification. The final average number of the four sections was regarded as the data for each sample. And the data were presented as the number of neurons per high-power field. All the process was conducted by two pathologists blinded to the grouping.

### TUNEL staining and cell counting

The formalin-fixed tissues were embedded in paraffin and sectioned at 4 μm thickness with a microtome. The sections were detected for apoptotic cells by terminal deoxynucleotidyl transferase-mediated dUTPnickend labeling (TUNEL) method. In situ cell death detection Kit POD (ISCDD, Boehringer Mannheim, Germany) was used. The procedures ran according to protocol of the kit and the other references. Briefly, sections were deparaffinized, rehydrated, and washed with distilled water (DW). The tissues were digested with 20 g/ml proteinase K (Boehringer Mannheim, Mannheim, Germany) at room temperature for 15 min. Endogenous peroxidase activity was blocked by incubation in 0.3% hydrogen peroxide/methanol in PBS at 37°C for 30 min. The sections then were incubated with terminal deoxynucleotidyl transferase at 37°C for 60 min to add the dioxigenin-conjugatd dUTP to the 3’-OH ends of fragmented DNA. Anti-digoxigenin antibody peroxidase was applied to the sections to detect the labeled nucleotides. The sections were stained with DAB and counterstained slightly with hematoxylin. The positive cells were identified, counted and analyzed under the light microscope by an investigator blinded to the grouping. And apoptotic neuron counting was also restricted to the external granular lamina layer of the occipital cortex. Every third coronary section starting from 3.0 mm posterior to the optic chiasma was collected and a total of 10 sections from each animal were used for quantification. Six random high power fields (x400) in each coronary section were chosen bilaterally, and the mean number of apoptotic neurons in the six views was regarded as the data of each section. The final average proportion of apoptotic neurons of the sections was regarded as the data for each sample and the severity of brain damage was evaluated by apoptotic index, defined as the average percentage of TUNEL-positive neurons.

### Determination of the concentrations of MDA

The same part of the occipital cortex was obtained and immediately froze in liquid nitrogen until use. MDA content was measured by commercial kits (Nanjing Jiancheng Bioengineering Institute, Nanjing, China), according to the methods described by the assay kits. All standards and samples were run in duplicate. The tissue protein concentration was determined by using a standard commercial kit (Bio-Rad Laboratories, Hercules, CA, USA).

### Western blot analysis

The same part of the occipital cortex was obtained and immediately froze in liquid nitrogen until use. The frozen brain cortex tissue was mechanically lysed in 20 mM Tris, pH 7.6, which contains 0.2% SDS, 1% Triton X-100, 1% deoxycholate, 1 mM phenylmethylsulphonyl fluoride (PMSF), and 0.11 IU/ml aprotinin (all purchased from Sigma-Aldrich, Inc., St. Luis, MO, USA). Lysates were centrifuged at 12,000 × g for 20 min at 4°C. The protein concentration was estimated by the Bradford method with the Nanjing Jiancheng (NJJC) protein assay kit (Nanjing Jiancheng Bioengineering Institute, Nanjing, China). The samples (60 μ g per lane) were separated by 8% SDS-PAGE and electro-transferred onto a polyvinylidene-difluoride (PVDF) membrane (Bio-Rad Lab, Hercules, CA, USA). The membrane was blocked with 5% skimmed milk for 2 h at room temperature, incubated overnight at 4°C with 1:200 rabbit polyclonal anti- active Caspase-3 primary antibody (Abcam, UK), and β-actin (diluted 1:6,000 in PBST, Abcam, UK) was used as a loading control. After the membrane was washed for 10 min each for six times in PBST, it was incubated in the appropriate HRP-conjugated secondary antibody (diluted 1:400 in PBST) for 2 h. The level of Caspase-12 was detected according to the same procedure with 1:200 rabbit polyclonal anti- active Caspase-12 primary antibody (Abcam, UK) and β-actin(diluted 1:6,000 in PBST, Abcam, UK). The blotted protein bands were visualized by enhanced chemiluminescence (ECL) Western blot detection reagents (Amersham, Arlington Heights, IL, USA) and were exposed to X-ray film. Developed films were digitized with an Epson Perfection 2480 scanner (Seiko Corp, Nagano, Japan). Optical densities were obtained via Glyko Bandscan software (Glyko, Novato, CA, USA) and the Caspase-3/12 expression levels were normalized to β-actin.

### Brain water content measurement

Animals were killed by decapitation and exsanguination. After removal of the brains, the cerebellum and hemispheres were separated under an operating microscope. Both cerebral hemispheres were weighed to assess their wet weight. The hemispheres were dried for 48 hours at 100°C for the determination of the dry weight. Based on the gravimetric differences, brain water content was obtained through the following calculation: Brain water content (%) = (WW-DW)/WW × 100, where WW is the wet weight of double hemispheres (in g) and DW is the dry weight (in g).

### Statistical analysis

All data were presented as mean ± SD. SPSS 16.0 was used for statistical analysis of the data. All data were subjected to one-way ANOVA. Differences between experimental groups were determined by the Fisher’s LSD post-test. Statistical significance was inferred at P < 0.05.

## Results

### General observation and clinical evaluation

Among all the experimental groups, no SAH-related death was observed. None of the rabbits exhibited obvious neurological deficits. The activity scores of these experimental groups did not display a significant difference among all the experimental groups (P > 0.05, ANOVA, data not shown). The appetite scores of the rabbits in the SAH + hydrogen-rich saline group were lower than those observed in the SAH group, but the difference was not statistically significant (P > 0.05, data not shown). Moreover, after the two-time injection of blood, all the SAH models were qualified: obvious blood clot can be seen around the brain stem and in the basal cisterns in each of the rabbits 3 days after induced SAH.

### Nissl staining

**Figure 1:**
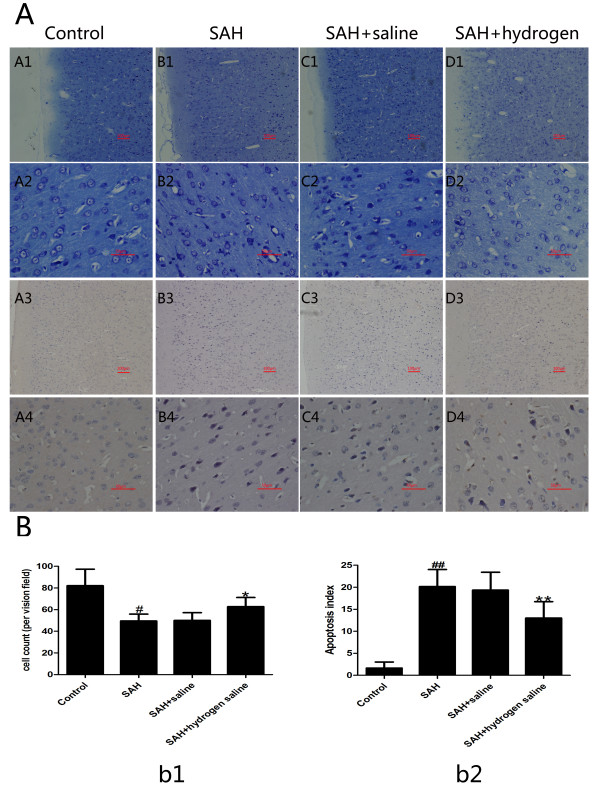
**Nissl and TUNEL staining (A) of cerebral cortex and cell counting (B) at 72 h following SAH.** (**A**) Representative slides of Nissl staining (A1-D2) and TUNEL staining (A3-D4) at two different magnifications (A1–D1, A3–D3: ×100; A2–D2, A4–D4: ×400). In the control group of Nissl staining (A1, A2), the neuronal cell outline was clear and the structure was compact with abundant cytoplasm and Nissl body. However, evident neuronal loss and neuronal degeneration were observed in the SAH group (B1, B2) and SAH + saline group (C1, C2). Cells arranged sparsely and the cell outline was fuzzy. The cells with eumorphism were significantly reduced. Compared with SAH or SAH + saline group, administration of hydrogen-rich saline substantially increased the proportion of survived neurons (D1, D2). TUNEL staining (A3-D4) showed that the rabbits in the control group display rare apoptotic neurons in the cortex (A3, A4), while obvious TUNEL positive neurons with intense nuclear stained as brown could be observed in the SAH group (B3, B4) or SAH + saline group (C3, C4). In contrast, the proportion of apoptotic neurons decreased significantly in the SAH + hydrogen-rich saline group (D3, D4). (**B**) Cell counts per visual field (×400) found in the slides with Nissl staining (b1) and TUNEL staining (b2). (b1) ^#^P < 0.05 compared with control group; *P < 0.05 compared with SAH or SAH + saline group. (b2) ^##^P < 0.05 compared with control group; **P < 0.05 compared with SAH or SAH + saline group.

Nissl staining was used to illustrate the morphology change of the cortical neurons (Figure 1, A1-D2). In the control group, rare injured neuron was detected (A1, A2). The visual field was full of clear and intact neurons, without edema around the cells. However, a significant proportion of neurons in the SAH (B1, B2) and the SAH + saline group(C1, C2) were damaged, exhibiting extensive degenerative changes including sparse cell arrangements, loss of integrity, shrunken cytoplasma, oval or triangular nucleus and swollen cell bodies. In contrast, the severity of neuronal degeneration in the SAH + hydrogen-rich saline group was evidently alleviated than that in the SAH or SAH + saline group (D1, D2).

### TUNEL staining

As is shown in Figure 1(A3-D4), few TUNEL-positive apoptotic neurons were found in the cortex of the control group (A3, A4). In the SAH (B3, B4) and SAH + saline (C3, C4) groups, the percentage of apoptotic neuron in the cortex increased substantially in comparison with that in the control group. However, compared with the SAH or SAH + saline group, administration of hydrogen-rich saline reduced neuronal apoptosis obviously (D3, D4). These results show that hydrogen-rich saline administration could alleviate neuronal apoptosis following SAH.

### Content of MDA

**Figure 2:**
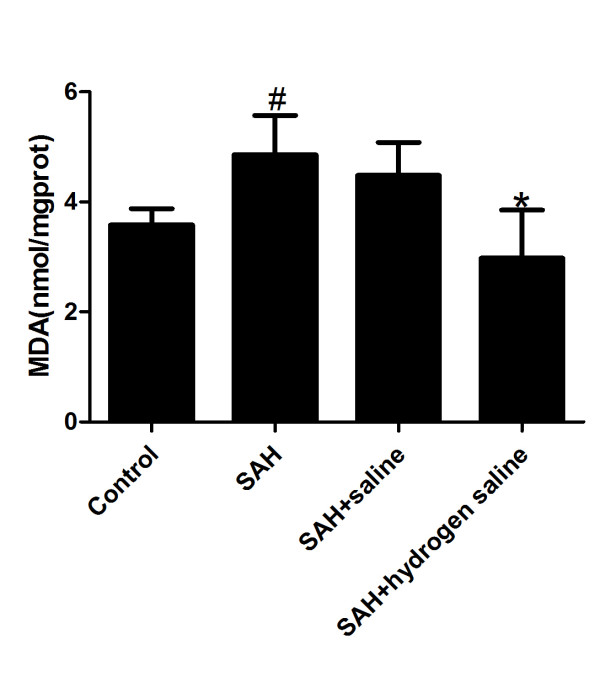
**Content of MDA.** MDA increased dramatically in the cortex following SAH. In contrast, hydrogen-rich saline administration reduced the level of MDA obviously after SAH. Bars represent the mean ± SE. ^#^P < 0.05 compared with control group; *P < 0.05 compared with SAH or SAH + saline group.

The content of MDA, which is the primary outcome, was detected in the control group and the experimental groups 72 hours after SAH. Compared with the control group, the content of MDA in SAH and SAH + saline group increased apparently. However, hydrogen-rich saline administration dramatically suppressed the production of MDA after SAH when compared with that in the SAH group (Figure 2).

### Caspase-12 and caspase-3 activity

**Figure 3:**
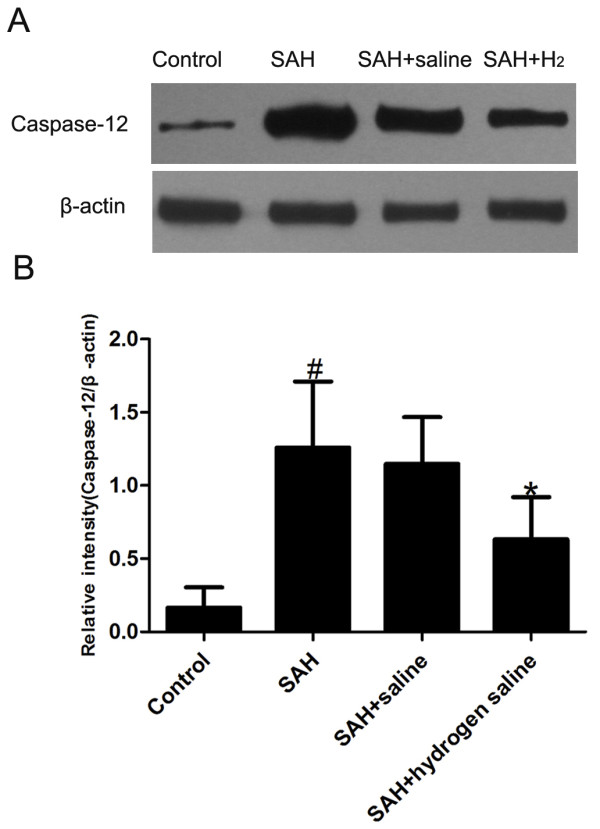
**Representative Western blots of caspase-12 activity in rabbit cortex in the experimental groups.** (**A**) Representative autoradiogram of the caspase-12 expression by Western blot. (**B**) Quantitative analysis of caspase-12 expression detected by Western blot. As is shown, caspase-12 was significantly increased in the SAH and SAH + saline groups compared with that in the control group. However, caspase-12 expression decreased obviously in the hydrogen-rich saline group compared with SAH or SAH + saline groups. Bars represent the mean ± SE. ^#^P < 0.05 compared with control group; *P < 0.05 compared with SAH or SAH + saline group.

**Figure 4:**
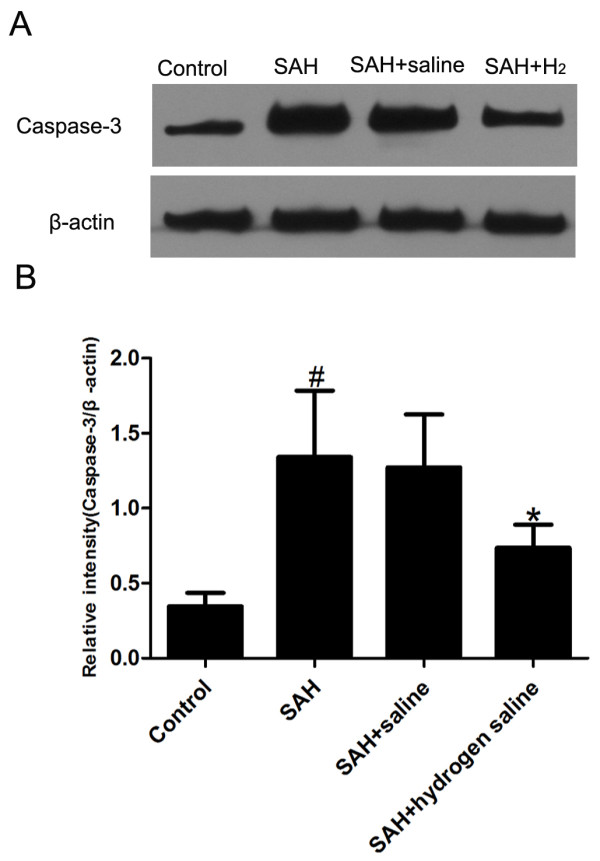
**Representative Western blots of caspase-3 activity in rabbit cortex in the experimental groups.** (**A**) The activity of caspase-3 detected by Western blot in the experimental groups. (**B**) Quantitative analysis of caspase-3 expression detected by Western blot. As is shown, the level of caspase-3 increased substantially in the SAH and SAH + saline groups compared with that in the control group. In contrast, caspase-3 expression decreased significantly in the SAH + hydrogen-rich saline group compared with that in the SAH or SAH + saline group. Bars represent the mean ± SE. ^#^P < 0.05 compared with control group; *P < 0.05 compared with SAH or SAH + saline group.

The activities of caspase-12 and −3 were measured at 72 h after SAH insult. As shown in Figure 3, SAH induced evident increase of caspase-12 in the cortex of SAH and SAH + saline group, while the level of caspase-12 decreased markedly in the SAH + hydrogen-rich saline group. Similarly, obvious increase of caspase-3 was observed in the cortex of SAH and SAH + saline group, and the administration of hydrogen-rich saline decreased the level of caspase-3 significantly (Figure 4).

### Brain water content measurement

**Figure 5:**
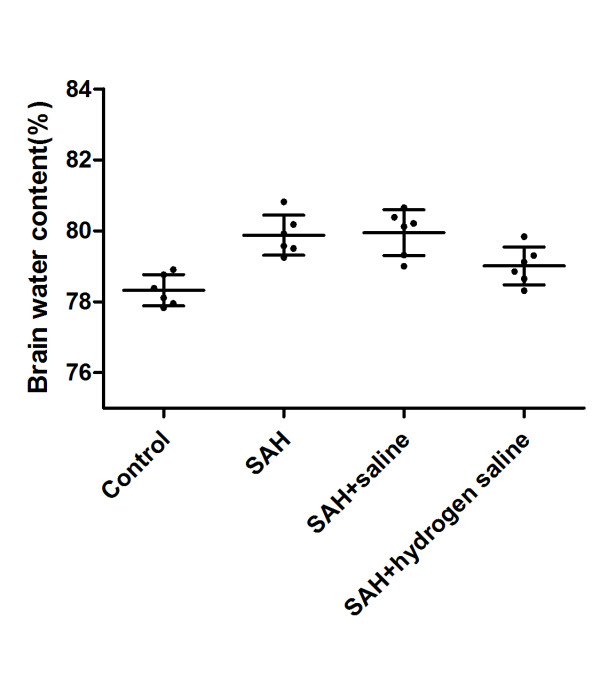
**Brain water content measurement.** As shown, brain water content increased distinctly in rabbits of SAH group and SAH + saline group, and hydrogen-rich saline treatment alleviated brain edema following SAH significantly. The values are expressed as mean ± SEM (n = 6 per group). ^#^P < 0.05 compared with control group; *P < 0.05 compared with SAH group.

Brain water content as an indicator of brain edema was measured at 72 h after SAH. As is shown in Figure 5, SAH caused significant increase of brain water content in the SAH and SAH + saline group. In contrast, the administration of hydrogen-rich saline reduced the brain water content markedly. These results suggested that hydrogen-rich saline treatment can significantly attenuate the development of SAH induced brain edema.

## Discussion

The main findings of this study are as follows: (1) SAH induced a significant increase of oxidative stress and evident brain edema, resulting in obvious brain damage in the rabbit SAH model; (2) Intraperitoneal administration of hydrogen-rich saline abated the level of oxidative stress and brain edema following SAH and, in the meanwhile, alleviated EBI following SAH. These findings suggest that hydrogen-rich saline could alleviate brain injury via decreasing oxidative stress and brain edema after SAH. Hence, we conclude that hydrogen-rich saline could be a potential therapeutic agent for EBI after SAH.

Oxidative stress arises from the strong cellular oxidizing potential of excess reactive oxygen species (ROS), or free radicals [28,29]. Plenty of ROS are generated after SAH and considerable evidences have confirmed that oxidative stress, which is the focus of this study, is an important mediator of brain injury after SAH [12]. Triggered by clot-derived hemoglobin(Hb), free radicals, including ·O^-^_2_·, H_2_O_2_ and ·OH, explode subsequently. ·O^-^_2_· is produced by hemoglobin auto-oxidation and consequent dismutation of two ·O^-^_2_· forms H_2_O_2_, and the latter is the source of highly reactive ·OH in the reaction catalyzed by ferric ion [30]. ·OH is the strongest of the oxidant species and reacts indiscriminately with nucleic acids, lipids and proteins to produce strong cytotoxic effect [20,31]. The production of ·OH from extravasated hemoglobin [32], mitochondria disruption [33] and the disruption of the intrinsic antioxidant system [34] have all been proved in experimental or human SAH. It has been demonstrated that explosion of free radical species after SAH caused oxidative brain injury [35], and antioxidative treatments alleviated brain damage and improved the neurological deficits in a rat model [36].

Lipid peroxidation is mediated by superoxide, such as ·OH, ONOO^-^, as well as H_2_O_2_, resulting in structural alterations of membranes and functional impairment of cellular components. Malondialdehyde (MDA), the end product of lipid peroxidation of polyunsaturated fatty acids in cellular membranes, was identified as a reliable marker of oxidative stress, which reflects the damages of the cell indirectly [37]. In agreement with previous reports [25,38], we confirmed in the present study that experimental SAH elevated MDA significantly in the brain cortex after SAH. And the administration of hydrogen-rich saline decreased MDA dramatically in comparison with that in the SAH or SAH + saline group.

Caspases, or cysteine-aspartic proteases or cysteine-dependent aspartate-directed proteases are a family of cysteine proteases that play essential roles in apoptosis (programmed cell death), necrosis, and inflammation [39]. Caspase-3 is one member of the caspase family and it plays a central role in the execution-phase of cell apoptosis. It has been reported that the expression of caspase-3 was intensified in the cortical neurons after SAH [40] and its inhibition could reduce the neuron loss. Caspase-12 is recognized to be essential for the endoplastic reticulum (ER) stress-induced apoptosis. The ER is sensitive to alterations in homeostasis from a variety of stimuli, such as glucose deprivation, perturbation of calcium homeostasis, and exposure to free radicals. Excessive stress to the ER results in apoptosis. It has been demonstrated that during apoptosis induced by ER stress, caspase-12 is localized to the ER and gets activated [41]. Caspase-12 is considered as an upstream caspase that might activate caspase-3 [42], and calcium–calpain–caspase-12–caspase-3 cascade was also suggested to play a role in ER-mediated apoptosis [43]. In the present study, we analyzed the level of caspase-3 and caspase-12 in the brain cortex following SAH. Our data showed that caspase-3 and caspase-12 increased significantly in the SAH and SAH + saline group but decreased dramatically in the SAH + hydrogen-rich saline group. These result are in line with the previous study in brain ischemia [44] and spinal cord injury [24]. Therefore, we can postulate that ER stress plays a pivotal role in the neuron death after SAH, and it might be a promising filed to studying the concrete protective mechanism of hydrogen in vivo.

Delayed global edema has been shown to be an independent predictor of death [13]. Cerebral edema, which can lead to increased intracranial pressure (ICP), brain herniation, irreversible brain damage or even death, is a significant clinical consequence following SAH. Brain edema is generally classified into cytotoxic or vasogenic edema [45]. Cytotoxic edema is defined as a cellular swelling with fluid accumulating within the cell and with the typical instance of astrocyte swelling [46]. Vasogenic edema is characterized by the breakdown of the BBB, resulting in the increased fluid accumulation around cells [47]. It has been postulated that altered expression of water channels aquaporins (AQPs), development of BBB disruption [48], active substances derived from blood, as well as secondary events after SAH, such as raised ICP [49] and hypertension are involved in the pathogenesis of brain edema. Badaut et al. [50] found a marked increase in AQP1 and AQP2 protein expression 24 hours after SAH in the human neocortex [51]. One of the early indications of brain injury after SAH is the alteration of BBB permeability [48]. In patients with SAH, classic vasogenic edema, a direct result of BBB breakdown, has been proved, which has also been shown in experimental models [52]. In addition, it has been proved that early dysfunction of BBB contributes to brain edema [53] which expands brain volume and prolongs elevated ICP values after SAH [54]. Consequently, there is a resultant rise in ICP, which further reduces cerebral blood flow, leading to further ischemia [49]. These bring more damage to the BBB. A vicious circle like this never ends. In this study, we found that the brain water content increased obviously after SAH and administration of hydrogen-rich saline abated brain edema significantly. This accords with what was reported in the brain trauma model by Ji et al. [55].

It is believed that oxidative stress is closely linked with brain edema via the discruption of BBB. Oxidative stress is considered as a major underlying cause of BBB injury. Oxidative stress-mediated disruption of BBB was shown in experimental models in vivo [56] or in vitro [57]. It has been demonstrated that alcohol-induced loss of BBB integrity is associated with increased production of ROS [58]. In an in vivo study, Katsu,et al. found that hemoglobin-induced oxidative stress contributes to matrix metalloproteinase activation and BBB dysfunction[59].

Hydrogen is a highly diffusible gas, which can easily penetrate the blood – brain barrier by gaseous diffusion. And hydrogen can also penetrate biomembranes smoothly to diffuse into the cytosol, nucleus and mitochondria. This is particularly important, as mitochondria, the primary site of generation of reactive oxygen species, is notoriously difficult to target. Ohsawa, et al. [20] reported that hydrogen can target intracellular sources of reactive oxygen species and inhibit reperfusion-induced oxidative damage by selectively scavenging ONOO^-^ and ·OH, the strongest of the oxidant species which reacts indiscriminately with nucleic acids, lipids and proteins. Therefore, H_2_ could selectively react with the ·OH to produce water, and did not react with other ROS that possess physiological functions. In this aspect, H_2_ is evidently superior to some other antioxidant with strong reductive reactivity, which can increase mortality, possibly by affecting essential defensive mechanisms [60]. This is immensely advantageous for medical treatment as the use of H_2_ would not bring serious side effects. Up till now, increasing evidence has proved that H_2_ can be used as an effective antioxidant therapeutic owing to its ability to decrease cytotoxic ROS [22,25,55]. In this study, we find that, the administration of hydrogen-rich saline decreased oxidative stress (MDA) and caspase-3/12 distinctly, which is in accordance with the previous reports. Meanwhile, hydrogen-rich saline abates the brain edema induced by SAH significantly. Correspondingly, the brain injury was alleviated in the SAH + hydrogen-rich saline group, and all the results in the present study were basically in accordance with the previous related reports [25,61].

With regard to the animal model, till now, a number of SAH models have been applied, and three of them are commonly seen: the intracranial endovascular perforation model, the blood injection into the cisterna magna model, and the blood injection into the prechiasmatic cistern model. However, controversy exists regarding which method of selection is appropriate for different animals and the drawbacks of each of the models [62]. Blood injection into the cisterna magna is the most frequently applied experimental SAH method in rabbits and both the one- and two-hemorrhage models are used to make rabbit SAH model. In our previous study, it was proved that the two-hemorrhage model in rabbits is more appropriate than the one-hemorrhage model for the research on SAH [63]. Consequently, the two-hemorrhage rabbit SAH model was used in the present study. However, like other models, the two-hemorrhage rabbit SAH model could not imitate the clinical SAH ideally as well. None of the rabbits died till 72 hours following SAH and evident neurological deficits could rarely be observed. Admittedly, there are many differences between the two-hemorrhage rabbit SAH model in this study and the clinical SAH. Hence, objectively, the SAH model in this study still needs further improvement, though obvious brain injury was found following the two-time SAH.

In addition, there was no statistically significant difference in clinical evaluation of the rabbits among the groups in this study, while evident neurological improvement was observed in the hydrogen treated hypoxia–ischemia rat model [25]. In effect, there are fewer behavior tests devised for the rabbit SAH model, and the differences in model and the scoring system could bring about inconsistent findings. As a result, we extrapolate that, to some extent, the neurobehavior evaluation system in the present experiment is not sensitive enough to discriminate the neurological difference in different groups. More sensitive neurobehavior tests for rabbit are necessitated in order to render the results more accurate and scientific, with which Hyojin Jeon et al. share the same opinion [64].

## Conclusion

Here, we demonstrated that hydrogen-rich saline could alleviate early brain injury via suppressing oxidative stress and brain edema after SAH. Hence, it may be a potential therapeutic agent for early brain injury following SAH. However, the molecular mechanisms of hydrogen underlying its marked effects remain elusive, and there is limited information on hydrogen related pathway in vivo. Therefore, much work remains to be conducted to elucidate this novel area, including the concrete mechanism and the outcome in the long term.

## Abbreviations

EBI, Early brain injury; SAH, Subarachnoid hemorrhage; H2, Hydrogen; BBB, Blood brain barrier; ROS, Reactive oxygen species; H2O2, Hydrogen dioxide; ·OH, Hydroxyl radical.

## Competing interests

The authors declare that they have no competing interests.

## Authors’ contributions

YWC and ZL participated in making experiment animal model. ZML and MCY guided the experimental procedures. SXJ and SJX participated in the design of the study and the writing of the manuscript. All authors read and approved the final manuscript.
